# The role of γδT lymphocytes in atherosclerosis

**DOI:** 10.3389/fimmu.2024.1369202

**Published:** 2024-05-07

**Authors:** LiMin Xu, Fanfan Chen, Wei Fan, Suguru Saito, DuoYao Cao

**Affiliations:** ^1^ Department of Neurosurgery, Shenzhen Entry-Exit Frontier Inspection Hospital, Shenzhen, China; ^2^ Department of Neurosurgery, Shenzhen Key Laboratory of Neurosurgery, The First Affiliated Hospital of Shenzhen University, Shenzhen Second People’s Hospital, Shenzhen, China; ^3^ Karsh Division of Gastroenterology and Hepatology, Cedars-Sinai Medical Center, Los Angeles, CA, United States; ^4^ Department of Biomedical Sciences, Cedars-Sinai Medical Center, Los Angeles, CA, United States

**Keywords:** γδT cells, atherosclerosis, αβT cells, metabolism, IPSC

## Abstract

Atherosclerosis poses a significant threat to human health, impacting overall well-being and imposing substantial financial burdens. Current treatment strategies mainly focus on managing low-density lipids (LDL) and optimizing liver functions. However, it’s crucial to recognize that Atherosclerosis involves more than just lipid accumulation; it entails a complex interplay of immune responses. Research highlights the pivotal role of lipid-laden macrophages in the formation of atherosclerotic plaques. These macrophages attract lymphocytes like CD4 and CD8 to the inflamed site, potentially intensifying the inflammatory response. γδ T lymphocytes, with their diverse functions in innate and adaptive immune responses, pathogen defense, antigen presentation, and inflammation regulation, have been implicated in the early stages of Atherosclerosis. However, our understanding of the roles of γδ T cells in Atherosclerosis remains limited. This mini-review aims to shed light on the characteristics and functions of γδ T cells in Atherosclerosis. By gaining insights into the roles of γδ T cells, we may uncover a promising strategy to mitigate plaque buildup and dampen the inflammatory response, thereby opening new avenues for effectively managing this condition.

## Introduction

Atherosclerosis contributes significantly to coronary artery disease, a leading cause of death worldwide (1). At its core, an imbalance in lipid metabolism leads to the formation of cholesterol-laden macrophages (foam cells) that are present in artery walls and greatly contribute to the development and rupture of atherosclerotic plaques (2). a diverse array of immune cells, including macrophages and T cells, infiltrate the intima of the plaque, playing a significant role in the progression of atherosclerosis. In many instances, the presence of lipid abnormalities leads to the apoptosis of endothelial cells, causing the release of inflammatory cytokines that attract circulating immune cells like monocytes to the sites of inflammation (3). Furthermore, T cells accumulate in the adventitia, particularly in arterial segments during the progression of atherosclerosis (4). These immune cells that are attracted to the site attempt to clear the apoptotic cells, but they encounter an abundance of surrounding lipids. Consequently, they uptake these lipids, leading to the formation of foam cells. These foam cells, in turn, continue to attract more immune cells, further contributing to the buildup of the atherosclerotic plaque.

T cells are characterized by surface markers, specifically the T-cell receptor (TCR), which plays a crucial role in adaptive immunity. Most T cells in humans are called αβ T cells, which can further be subdivided into subsets like CD4 T cells and CD8 T cells. These two subsets have been found to play a role in the progression and regression of atherosclerosis. Recent findings suggest that CD4 T cells can recognize peptides derived from apolipoprotein B in atherosclerosis models (5–7). On the other hand, CD8 T cells show a higher prevalence in the circulating blood and atherosclerotic lesion areas (8, 9). Compared to αβ T cells, γδ T cells express a unique TCR consisting of gamma and delta chains, which grants them diverse capabilities in engaging both innate and adaptive immune responses. γδ T cells represent a subset of T lymphocytes that comprise a relatively small fraction of peripheral blood (1%–5% of circulating lymphocytes) (10). However, they form the predominant subset of T cells residing in mucosal tissues and skin, serving a unique and crucial role in immune defense that sets them apart from other lymphocytes. Recent findings suggest a pathogenic role of γδT cells in the early stages of atherogenesis in ApoE KO mice. These cells produce IL-17 instead of INF-γ, resulting in elevated circulating neutrophils (11). However, our understanding of the specific functions of γδ T cells, particularly their roles in the innate and adaptive immune responses in atherosclerosis conditions, remains limited. In this mini-review, we aim to uncover the roles of γδ T cells in atherosclerosis and explore potential therapeutic pathways utilizing γδ T cells for the treatment of atherosclerosis.

## T lymphocytes in atherosclerosis

Macrophages have been the predominant focus of immune cell research in the context of atherosclerosis formation over the past decades. These cells are essential in clearing apoptotic cells via efferocytosis and digestion in a normal lipid environment. However, disrupted lipid metabolism of innate immune cells (e.g., macrophages) could form foam cells in artery walls, a key contributor to atherosclerotic plaque development (2, 12, 13). When innate immune cells cannot effectively clear accumulated lipids in the lesion area, foam cells will attract additional adaptive immune cells to the site. In the later stages of atherosclerosis, the influx of adaptive immune cells, particularly T lymphocytes (αβT and γδT lymphocytes), participate in the inflammatory response in the plaque area.

αβ T cells are the predominant type of lymphocyte in both murine and human peripheral circulation. They can be further categorized into two major subsets based on their cell surface markers: CD4+ and CD8+ T cells. In atherosclerosis conditions, these subsets of αβT cells play crucial roles in the immune response, contributing differently to the overall detection and defense against lipid abnormalities in the body. CD4+ T cells, which represent the major population of αβT cells, have also been identified in atherosclerotic plaques. Th1 and Th17 cells, both subpopulations of CD4+ T cells, have been recognized as pro-atherogenic, whereas Th2 cells function in an anti-atherogenic capacity (7, 14). In mouse studies, Th1 cells within plaques exhibit high CC-chemokine receptor 5 (CCR5) expression and robustly produce pro-inflammatory cytokines, including IFNγ, IL-2, TNF, and the T-bet transcription factor. These factors can potentially stimulate the production of pro-inflammatory macrophages, thereby amplifying the inflammatory response (15–17). Compared with Th1 cells, Th2 cells are generally regarded as anti-inflammatory in atherosclerotic conditions. Clinical studies have demonstrated that individuals with a higher number of Th2 cells in peripheral blood mononuclear cells (PBMCs) exhibit a lower burden of subclinical atherosclerosis, as indicated by reduced common carotid intimal media thickness, in comparison to those with lower numbers of Th2 cells (18, 19). Furthermore, Th2-secreted cytokines, such as IL-5 and IL-13, have exhibited an atheroprotective role in both human and murine studies (20–22). Th17 is another subpopulation of CD4+ T cells identified as a major source of IL-17 secretion and exhibits distinct plasticity in various inflammatory contexts (23–25). Most studies have demonstrated that IL-17A is a pro-atherogenic cytokine in Apoe-/- mice studies (26–28). However, some results indicate that IL-17 may have opposing effects or no significant impact on atherosclerosis (27–30). Therefore, the roles and functions of Th17 cells in atherosclerosis need further exploration.

Furthermore, there is a divergence in research findings regarding the contribution of Treg cells to the advancement of atherosclerosis. Treg cells are known to release IL-10 and TGF-β, both of which have exhibited a protective effect on the progression of atherosclerosis, as demonstrated in both animal and clinical studies (31, 32). They can reduce atherosclerosis by modulating lipoprotein metabolism (33). However, it has been observed that when Treg cells lose FoxP3 expression, they may transform into T follicular helper (Tfh) cells, potentially intensifying the progression of atherosclerosis (34, 35). Additionally, another subset of CD4+ T cells, the Natural Killer T (NKT) cells, has been found to play a pro-atherogenic role in mouse models (36–38). The roles of other T cell subpopulations, such as Th9 and Th22, in atherosclerotic conditions remain unclear.

CD8+ T cells are prominent participants in antiviral and antitumor responses.

Notably, in the context of atherosclerosis, both patients and mouse models have demonstrated the accumulation of CD8+ T cells. These CD8+ T cells are known to secrete IFNγ, which can trigger inflammation and recruit monocytes, thus accelerating the atherosclerotic condition. This, in turn, leads to an enhanced presence of CD8+ T cells in both the circulation and atherosclerotic plaques (8, 39, 40). Furthermore, single-cell RNA sequencing data from the progression of human atherosclerotic plaques revealed two distinct γδ T cell clusters expressing TRGC1, TRGC2, and TRDC. Interestingly, this expression profile is similar to that of CD8 T cells, suggesting a potential exacerbation of atherosclerosis by these γδ T cell clusters (41).

## The roles and functions of γδ T lymphocytes in innate and adaptive immune responses

γδ T cells are yet another subset of T lymphocytes characterized by the presence of the γδ T cell receptor (TCR) on their cell surface. Although only a small population of these cells is found in peripheral blood (1%–10% of CD3^+^ T cells) (42), this type of cell constitutes the major subset of resident T cells in mucosa and skin. It plays a distinct role in immune protection compared with other lymphocytes (43). γδ T cells are particularly enriched in epithelial tissues, such as the reproductive tract, skin epidermis, and gastrointestinal tract, responding to potential danger or cellular stress signals. γδ T lymphocytes are composed of several subsets. In human or higher primates, gamma delta T cells are categorized based on TCRdelta usage, denoted by Vδ1+, Vδ2 + and Vδ3, etc (44, 45). In murine models, classification is determined by TCRgamma usage, indicated by Vγ1, Vγ4, Vγ5, Vγ6, and Vγ7 (46–50).

**Figure 1:**
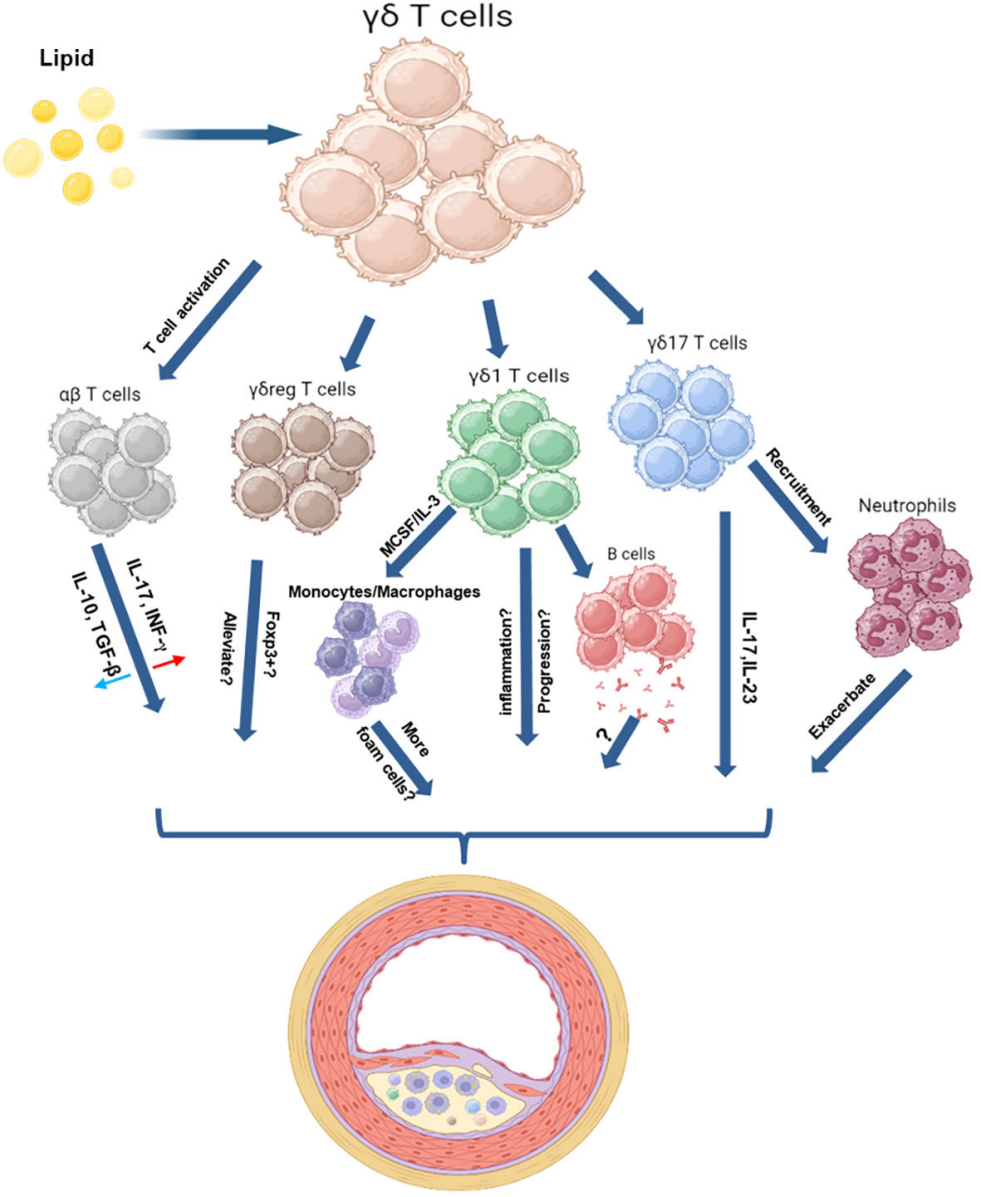
Schematic Illustration of the Roles of γδ T Cells in a High Lipid Environment. These schematic figures aim to illustrate the potential interactions between γδ T cells and other immune cells in a high-lipid environment. γδ T cells have proven their capacity to offer immune defense against bacterial and tumor threats. These cells perform various functions upon activation, including aiding B cells in antibody production, activating αβ T cells, promoting monocyte differentiation, recruiting neutrophils, and supporting tissue repair—an essential process for wound healing. In the context of atherosclerosis, γδ T cells may exhibit similar actions. Moreover, γδ T cells possess the capability to polarize into various subpopulations, including γδ1, γδ17, and γδreg, each playing distinct roles based on environmental stimuli. γδ17 cells, known for producing inflammatory molecules like IL-17 and IL-23, might accelerate atherosclerotic lesion formation. However, information about other subsets, such as γδ1 or γδreg cells, and their involvement during plaque formation is currently limited. The figure was created with BioRender.com.

As T lymphocytes, γδ T cells also have multiple functions. These cells play different roles in the immune response, such as cytokine production, antigen presentation, killer cell activity enhancement, and immune cell regulation (43). In contrast to αβT cells, γδT cells are not limited by APCs, which have the ability to recognize danger signals and then activate targeted cells (51, 52). Crowley et al. found that mouse γδT cells could recognize MHC IB antigens, such as T10 and T22 (53). In a human study, γδT cells, such as APCs, can also directly activate CD8^+^ γδT cells (54); therefore, γδT cells may trigger the immune response without any help from APCs and recruit other immunocytes to inflammation sites. When infection occurs, γδT cells will secrete cytokines (IFN-γ, IL-17, and others), thus promoting the recruitment of neutrophils to participate in the early stage of inflammatory responses (55). In addition to differences in cytokine repertoire, γδT cells exhibit diversity in homing and antibody production, such as migration to lymph node follicles, to help B cells by promoting antibody production in B cell follicles (56–58). In addition, various subsets of γδT cells have shown anti-inflammation and immunoregulatory activities as well as repair functions (43) (**Figure 1**).

Similar to αβ T cells, γδT cells can differentiate into γδ1, γδ2, γδ17, and others (43, 59) (**Figure 1**). Moreover, unlike other IL-17-producing cells that require initiation γδT cells can directly secrete IL-17 under certain inflammatory conditions (60). Roark et al. demonstrated that IL-17-producing γδT cells could differentiate and develop differently than Th17 cells to mount a quick response for protection against infection (61). Interestingly, responses of IL-17-producing cells are important for the host defense against microorganisms, particularly extracellular bacteria (62, 63). IL-17, produced by γδT cells, may trigger a positive feedback loop that further attracts Th17 and Th1 cells, dendritic cells, and neutrophils, amplifying host inflammatory responses. Moreover, unlike other IL-17-producing cells that require initiation, γδT cells can directly secrete IL-17 under certain inflammatory conditions (60). γδ T cells emerge as the principal reservoir of IL-17-producing cells, promptly engaging with antigens within mucosal tissues to fortify the body’s defense against infections. These results suggest that γδ T cells have great potential in antigen recognition and pathogen elimination, potentially fulfilling a distinctive role within the immune system.

Several studies have demonstrated that γδT cells, as the body’s first barrier, play a vital role in the mucosal immune response (64). In a mouse model infected with Streptococcus pneumoniae, the number of γδ T cells significantly increased in the lungs at 3, 6, and 12 hours post-infection. However, the recruitment of neutrophils sharply declined in TCR-Vγ4-/- mice. The bacterial clearance ability was impaired in TCR-Vγ4-/- mice compared to WT mice. This result demonstrates the critical role of γδT cells in neutrophil-mediated host defense against S. pneumoniae infection (65). In a study of oral *Yersinia pseudotuberculosis*, bacteria presented earlier invasion of the liver and spleen in γδT cell-deficient mice compared to WT mice (66). In addition, some studies found that γδ T cells were the predominant IL-17-producing cells that eliminated bacteria-induced pathogens, such as *E. coli* or *S. aureus.* γδT cells were found to be the primary producers of IL-17 after *E. coli* infection; antibody depletion of γδT cells led to a decline of IL-17 production and less neutrophil infiltration to the peritoneum (67). Cho et al. found that γδT cell-deficient mice were much more susceptible to *S. aureus* infection and presented impaired neutrophil recruitment than WT mice. Furthermore, our previous result showed that γδT cells, especially γδ17 cells, play an essential role in *S. aureus-*induced chronic mastitis (68). Interestingly, γδT cells could directly recognize lipoteichoic acid (LTA) by the scavenger receptor CD36 (69). Thus, Long-chain fatty acids may also activate γδT cells via CD36 receptor ligands (70, 71). These results demonstrated that γδT cells might protect the body against pathogen invasion and provide protection in the early stage of infection. Interestingly, certain bacteria could also promote the progression of atherosclerosis (72). However, the role of γδT cells during this process remains unknown, potentially providing a new direction for atherosclerosis research.

γδT cells have diverse functions in physiological and pathological processes during infection. These cells release cytotoxicity effector molecules, such as perforin and granzyme, to kill infected cells and to directly or indirectly activate immunocytes and epithelial cells to participate in pathogen elimination (73, 74). γδT cells also secrete bacteriostatic or lytic molecules to directly clear pathogens in mucosal immunity (59). In addition, a variety of pathogens could induce γδT cells to produce different cytokines, for instance, TNF-α and IFN-γ were the major secretions in viral or intracellular bacterial infection; IL-17 was the main product in extracellular bacterial or fungal infection; IL-4, IL-5 and IL-13 were the primary cytokines produced upon extracellular parasite stimulation (43, 59). Additionally, γδT cells can produce immunosuppression cytokines such as TGF-β or IL-10 to regulate innate or adaptive immunity and promote tissue repair and epithelial cell regeneration (59, 75). Previous results indicated that γδ T cells could directly mediate host infection and bridge innate and adaptive immune responses (**Figure 1**).

γδ T cell also demonstrate their reparative role by playing a pivotal role in tissue repair by producing cytokines and growth factors. Normal wound closure was restored by supplementing rapamycin-treated mice with skin γδT cells released elements (76). In corneal friction impairment, CCR6+ IL-17+ γδ T cells rapidly migrate to the basal layer of the corneal stratum to contribute to epithelial healing; however, the process of epithelial healing was notably impaired in TCRγδ-deficient mice (77). γδT cells are also involved in adaptive immunity-mediated inflammation. In an inflammatory bowel disease (IBD) mouse model, γδT cells exacerbate colitis in TCRγδ^-/-^ mice probably by promoting Th1 and Th17 differentiation (78). γδT cells could also cooperate with γδT cells to participate in the inflammatory response and migrate to lymph nodes to help B cells produce antibodies for pathogen elimination (59) (**Figure 1**).

## γδ T cells in atherosclerosis

αβ T cells primarily participate in adaptive immune responses, recognizing peptides through antigen-presenting cell MHC. On the other hand, the recognition process of γδT cells is independent of MHC and includes non-peptide antigens like phospholipids and organic molecules. Additionally, γδT cells exhibit the ability to process environmental information more rapidly than αβ T cells. These findings suggest that under atherosclerotic conditions, γδT cells may exhibit enhanced efficiency in lipid processing, highlighting the need for further exploration.

In high lipid environments, γδ T cells have been observed to promote inflammation and insulin resistance significantly. This is achieved through the upregulation of cytokine production (such as IL-6, TNF-a, etc.) and the recruitment of inflammatory macrophages in obesity mouse model (79). In ApoE/γδ T cells double knockout (DKO) mice, a substantial reduction in circulating neutrophils was observed when these DKO mice were on a Western diet. Notably, the expansion of inflammatory monocytes and splenic Th1 or Th17 lymphocytes remained unaffected (11). Neutrophils show a higher abundance in early atherosclerotic lesions compared to more advanced plaques. Also, Neutrophils are the major source of IL-23, they could collaborate with IL-23R+ γδ T cells, collectively contributing to the initiation of inflammation in the vessel wall (80, 81). These findings suggest a significant connection between γδ T cells and neutrophils in atherosclerosis, indicating a potential therapeutic target for treatment.

Innate immune cells play a crucial role in the early stages of responding to high lipid. When an excess of lipids is present, the innate immune cells and endothelial cells, acting as the initial line of defense, promptly release pro-inflammatory cytokines like IL-1β, IL-6, and TNF-α at inflammatory sites. This action is followed by the recruitment of additional lymphocytes, such as Th1 cells, which accelerate the progression of atherosclerosis. Conversely, during the stages of atherosclerosis regression, innate immune cells can express ACE (angiotensin-converting enzyme), IL-4, and IL-10 and TGF-β to attract reparative lymphocytes like Th2 cells. Additionally, they reprogram macrophage metabolism by modulating ACE expression towards oxidative phosphorylation (OXPHOS), aiming to mitigate atherosclerosis (3, 82–85). γδ T cells are regarded as members of the innate immune system, playing a crucial role in innate immune recognition and bridging innate and adaptive immunity. Consequently, they could be a promising target for the treatment of atherosclerosis.

Reparative macrophages play a pivotal role in the regression of atherosclerosis. However, their abundance is often compromised by the heightened presence of IL-17 within the atherosclerotic milieu. IL-17 has been identified as a key factor in inhibiting the polarization of M2 macrophages while concurrently stimulating the proliferation of M1 macrophages. Studies utilizing murine models of chronic trauma have underscored the potential of IL-17-neutralizing antibodies in bolstering the population of M2 macrophages (86, 87). Moreover, γδ17 cells emerge as prominent contributors to the IL-17 pool, particularly during the progression of atherosclerosis, compared to other γδ T cell subsets. This prevalence of γδ17 cells in the initial stages of atherosclerosis is implicated in impeding the recruitment and activation of reparative macrophages. Furthermore, in the context of metabolic disorders such as obesity, γδ17 cells have been observed to exacerbate inflammation and insulin resistance through heightened cytokine production, including IL-6 and TNF-α, and the recruitment of proinflammatory M1 macrophages (79). Additionally, investigations in psoriasis mouse models have revealed a potential link between Ly6C high monocytes/macrophages and the accumulation of γδ17 cells mediated by the secretion of IL-23 and IL-1β (88, 89). These findings collectively suggest a mechanistic association between γδ17-derived IL-17 and the dampening of M2 macrophage polarization, thereby perpetuating atherosclerosis progression.

On another front, the role of γδ T cells in antigen presentation is noteworthy. These cells possess a broader antigen recognition repertoire compared to conventional αβ T cells, enabling them to respond to diverse non-peptide antigens. In conditions such as sepsis, however, the antigen-presenting functions of γδ T cells may be compromised, leading to reduced activation of CD4+ T cells. Nevertheless, in healthy individuals, γδ T cells typically maintain their APC functionality (89, 90). Recent studies have shown that the infusion of allogeneic Vδ2 T cells can increase the proportions of both CD4+ and CD8+ T cells in the peripheral blood of most patients (90). Additionally, CD1 molecules, a family of cell surface proteins responsible for presenting lipid antigens to T cells, have been implicated in antigen presentation (91). Notably, CD1 proteins, including CD1a, -b, -c, and -d, are highly expressed in atherosclerotic plaques (91, 92). These lipid antigens presented by CD1 encompass a diverse array, ranging from foreign lipids unique to specific microorganisms to common mammalian self-lipids (93). Human γδ T cell receptors (TCRs) have been found to recognize CD1 molecules via Vδ1+ or Vδ3+ subsets and can respond to various presented phospho- and glycolipids (90). This suggests that γδ T cells may play a role in recognizing and presenting foreign lipids to αβ T cells during the formation of atherosclerosis. These findings highlight the potential of γδ T cells as APCs capable of promoting the proliferation of αβ T cells, hinting at a therapeutic avenue for mitigating atherosclerosis.

In the landscape of atherosclerosis, cytokines are broadly categorized as either pro- or anti-atherogenic. Pro-atherogenic cytokines like IL-17, IL-1β, and IL-6 exert significant influence on plaque formation, while anti-atherogenic cytokines such as IL-10, TGF-β, IL-5, and IL-13 have been associated with reduced plaque formation. During the progression stage, γδ T cells have been observed to increase notably in high lipid environments, releasing proinflammatory cytokines like IL-6, IL-1β, and IL-17, potentially exacerbating plaque vulnerability (94). Single-cell RNA sequencing data from both human and mouse studies have revealed an increased number of γδ T cells in adventitial artery tertiary lymphoid organs (ATLOs), exhibiting elevated expression levels of certain genes including *Cxcr6, Lgals1, Reep5* and *S100a6* (95). Consequently, in the context of atherosclerosis, γδ T cells may promote inflammation by releasing proinflammatory cytokines and chemokines, thus accelerating disease progression. Despite limitations in studying the subsets and functions of γδ T cells in atherosclerosis, their involvement in cardiovascular-related diseases such as myocardial infarction and myocardial ischemia has been extensively investigated (96–98). Studies have shown that γδ T cells are recruited to the myocardium after myocardial infarction in both humans and mice, acting as a major source of IL-17A, which promotes inflammation (97, 98). People have observed increased expression of CD69 in Tregs after myocardial infarction in patient samples. Knockout experiments in mouse models have revealed that the absence of CD69 dramatically increases IL-17+ γδ T cells, exacerbating inflammation and impairing cardiac function (99). In myocardial ischemia, depletion of IL-17A or γδ T cells has been shown to improve the survival rate of mice after early myocardial ischemia (100). These discoveries indicate that γδ T cells might be promising targets for treating cardiovascular conditions. Specifically, decreasing IL-17+ γδ T cells could potentially attenuate the advancement of cardiovascular inflammation.

In contrast, during atherosclerosis regression, γδ T cells, as part of the innate immune system, may detect lipids, releasing cytokines that attract regulatory cells to the inflammatory site, thereby reducing plaque expansion. In experiments involving high cholesterol treatment, γδ T cells have displayed higher activation levels of lipid digestion markers (ABCA1 and ACAT1/2) compared to αβ T cells (101). Additionally, in ruminants, γδ T cells play a crucial regulatory role in the immune system, spontaneously secreting IL-10 and proliferating in response to specific stimuli (102). Notably, IL-10 and TGF-β have been identified as major cytokines associated with atherosclerosis regression (103). Furthermore, studies on obesity have shown that cytokine levels such as IL-13 and IL-5 are significantly lower in γδ T-deficient obese mice compared to WT mice (104). These findings suggest a potential reparative role for γδ T cells during the process of atherosclerosis regression, further highlighting their importance in mitigating disease progression and promoting vascular health.

Recent finding has been shown that γδ T cells especially Vδ2 cells are activated, independent of MHC, by small lipid molecules, phosphoantigens (pAgs), which are derived from the mevalonate pathway (105). Additionally, specific lipid-related ligands, including apolipoprotein A1 (Apo-A1) and ATP synthase/F1-ATPase (recognized as a high-affinity apo A-I receptor), have been identified as ligands for the Vɣ9Vδ2 TCR on tumor cells, suggesting a potential role for γδ T cells in recognizing lipid molecules (106). In patients with Coronary Artery Disease (CAD), a lower absolute number of circulating γδ T cells has been observed. This may be attributed to an increase in Fas expression on the surface of γδ T cells in CAD patients, potentially mediating apoptosis (107). Above findings suggest that γδ T cells could recognize ApoA, which may positively correlate with HDL and have beneficial effects on cholesterol efflux, thereby promoting atherosclerosis regression. Investigating the connection between γδ T cells and apolipoproteins during atherosclerosis regression may offer new insights for treatment strategies.

Immunometabolism can reprogram cells according to their energy environment. αβT cells have been observed to utilize various metabolic pathways and metabolites that can modulate T cell proliferation, survival, differentiation, and function (108, 109). In contrast to αβT cells, our knowledge regarding the metabolism of γδT cells remains limited. In tumor microenvironments, γδ T cell subsets that produce either IFN-γ or IL-17 exhibit inherently distinct metabolic requirements (109, 110). Lopes et al. discovered that γδIFN T cells exhibit a high degree of glycolysis, similar to CD8+ T cells. Conversely, γδ17 T cells rely on oxidative phosphorylation (OXPHOS) and exhibit increased mitochondrial mass. Their study also identified two major transcription factors, NRF1 and Myc, which regulate OXPHOS and glycolysis (109, 110). In another study, glutamine has been identified as a crucial regulator in γδ17 T cells associated with skin inflammation (111). Fatty acids, which serve as a primary energy source to sustain the body’s daily requirements, can also induce reprogramming of T cell functions across various dimensions. As T cells cannot synthesize fatty acids internally, they rely on the abundant circulating fatty acids, which can interact with T cells and influence every aspect of their responses. The outcomes vary depending on the specific fatty acid the T cell is exposed to. Unsaturated fatty acids, prevalent in anti-inflammatory responses, contribute to an atheroprotective role. In contrast, saturated fatty acids, recognized as proatherogenic factors, tend to incite a more pro-inflammatory reaction during T cell activation (112). External fatty acids can impact the differentiation of Th17 cells by modifying T cell metabolism through acetyl-CoA carboxylase 1 (ACC1) (113). One human study revealed that adding palmitoleic acid to activated human T cells did not induce cytotoxic effects. However, it did reduce the production of IL-17A, IL-2, IFNγ, and TNF, while simultaneously decreasing the number of Treg cells (114). Polyunsaturated fatty acids (PUFAs) like EPA and DHA exhibit distinct anti-inflammatory properties, enhancing the proportion and cytokine levels of anti-inflammatory Th2 cells and Treg cells while reducing those of pro-inflammatory Th1 and Th17 cells in both vitro and vivo (112).

Despite the initial identification of γδ T cells in human atherosclerosis lesions, their precise role in the progression and regression of atherosclerosis remains unclear (11, 101, 115). Since the first identification of γδT cells in human atherosclerotic lesions, only a few studies have been conducted in mice investigating their involvement in atherosclerosis (11, 101, 115). Notably, during the early phases of atherogenesis, there was a marked increase in the numbers of γδT cells within the proximal aorta of ApoE-deficient mice compared to wild-type counterparts. This elevation was particularly pronounced in the aortic root and arch, where γδT cells constituted the predominant T cell population, coinciding with the most rapid lesion progression. These aortic γδT cells were identified as IL-17 producers but not IFN-γ (11). Interestingly, it was found that the intracellular cholesterol content in γδT cells significantly impacted their activation, proliferation, and effector functions (101). Additionally, γδT cells emerged as a major source of IL-17 in murine models, potentially regulating IL-17 production in atherosclerosis. Bone marrow-derived CD27-positive γδT cells promote atherosclerosis and influence plaque stability. This promotion occurs through their direct involvement in lesion inflammation and cell death, facilitated by the release of IFN-γ and perforin, ultimately expanding vulnerable plaques (116). Additionally, IL-23R+ γδ T cells are primarily concentrated in the aortic root, exhibiting substantial expression of IL-17 and GM-CSF. This implies a potential contribution to early atherosclerotic lesions and plaque necrosis initiation by activating macrophages through the secretion of IL-17A and GM-CSF (81). These observations strongly suggest a pro-inflammatory role for activated γδT cells in atherosclerosis. However, the precise role of these γδT cell subpopulations in atherosclerosis remains elusive, mainly due to limited investigative resources and postponed analyses of γδT cells in this context. Fortunately, recent advancements in sequencing technologies such as single-cell RNA sequencing and spatial transcriptomics provide new opportunities for a more comprehensive understanding of γδT cells, their subsets, and functions during the progression and regression of atherosclerosis.

## γδ T cells based therapy

γδ T cells possess the unique ability of independent antigen presentation, enabling direct infiltration into tumor environments. High levels of γδ T cells in cancer patients have been correlated with improved clinical outcomes across various malignancies. As mentioned above, Vδ2 cells are the dominant and most studied subset in human peripheral blood. Clinical applications of Vδ2 cells in cancer treatment have enhanced overall survival rates compared to control groups (117–123). Vδ1 cells also shown beneficial effects in skin, colon, and triple-negative breast cancers, improving clinical outcomes across various malignancies (124–126). γδ T cells exhibit rapid and effective target cell killing through the secretion of pro-inflammatory cytokines (such as IL-12) and cytotoxic molecules (granzymes and perforin), along with the expression of NK cell receptors, which hold promise against malignant cells (121, 127).

Based on their anti-tumor capabilities, numerous γδ T cell-based immunotherapies have been developed for cancer treatment. For Vδ2 T cells, approaches include using humanized anti-BTN3A antibodies to enhance their tumor-targeting ability (128, 129) or employing engineered tumor-γδ TCR bispecific antibodies (e.g., CD40, CD1D) to boost their cytotoxic efficiency (130, 131). Furthermore, there has been a surge in the utilization of modified γδ T cells, such as CAR- γδ T cells, which demonstrate enhanced cytotoxic potential compared to unmodified counterparts within the tumor microenvironment. Intriguingly, CAR-T Vδ2 cells maintain antigen-presenting potential *in vitro* (121, 132). Recently, there’s been increased focus on Vδ1 T cells, as they exhibit prolonged persistence rates *in vivo* (121). Studies have demonstrated that CAR Vδ1 T cells possess tumor-suppressive abilities, as evidenced in xenograft models of hepatocellular carcinoma and B cell lymphoma (133, 134). Moreover, researchers are utilizing retrovirus to implant TCRγδ onto αβ T cells, creating ‘T cells engineered with defined γδ TCRs’ (TEGs), which have demonstrated tumor suppression abilities in various models (135, 136). Although our understanding of γδ T cells reparative functions in inflammatory diseases is currently limited, we still can draw from anti-tumor methods to explore new approaches. For instance, atherosclerotic-γδ TCR bispecific antibodies and engineered reparative γδ TCRs could be promising avenues for atherosclerotic therapy.

Despite their promising potential, the utilization of γδ T cells in T-cell therapy faces challenges. Prolonged ex vivo expansion of γδ T cells can lead to a loss of anti-tumor efficacy due to γδ T cell exhaustion induced by long-term stimulations, including exposure to substances like ZOL and pro-inflammatory cytokines(IL-2, IL-15). This phenomenon, known as T cell exhaustion, presents a significant obstacle in harnessing the full therapeutic potential of γδ T cells for cancer therapy (137, 138). To address this challenge, Induced pluripotent stem cell (iPSCs) technology hold promise as they possess the ability for unlimited proliferation and multidirectional differentiation. Watanabe et al. showed that human peripheral blood mononuclear cells (PBMCs) were stimulated with IL-2 and zoledronate. Subsequently, these cells were transfected with a Sendai virus vector, resulting in γδT cell-dominant expression of exogenous genes, allowing approximately 70% of the cells to carry the TCRG and TCRD gene locus (137). Nobuyuki et al. successfully utilized human iPSCs to generate γδT cells. These iPSC-derived γδT cells have demonstrated potential applications in various cancers in an MHC-unrestricted manner (138). They identified distinctive features in these iPSC-derived γδT cells (iγδT) by using single-cell RNA sequencing. These cells exhibited lower CD2, CD5, and antigen-presenting gene expression. Surprisingly, CD7, Kit, and natural killer cell markers had higher expression. Additionally, iγδT cells expressed high levels of granzyme B and perforin (138). αβ Treg cells have been demonstrated to play a reparative role in the development of atherosclerosis (139), Tregs can also upregulate the expression of fatty acid transporter CD36 and PPAR-γ, potentially activating fatty oxidation to alleviate the progression of atherosclerosis (140). Similar to the αβ Tregs cell subset, enhancing γδreg cells could serve as a potential target for treating atherosclerosis. Additionally, freshly isolated human γδ T cells typically exhibit low expression of Foxp3 and CD25. However, after treatment with anti-human TCRγδ, the majority of expanded γδ T cells coexpressed Foxp3 and CD25 by day 5 (141). Interestingly, in a Type 1 diabetes (T1D) mouse model, Mohammad et al. utilized iPSC-Tregs (iTreg) to effectively suppress autoimmunity and prevent the destruction of insulin-secreting pancreatic beta cells. Furthermore, their study revealed that iTregs could reduce the expression of ICAM-1 in the diabetic pancreas, thereby inhibiting the production of the pro-inflammatory cytokine IFN-γ within the pancreas (142). Therefore, for future applications aimed at obtaining iPSC-induced γδreg cells, one could potentially treat the cells with specific molecular supplements, such as anti-human TCRγδ and other anti-inflammatory cytokines, to induce differentiation into γδreg cells for the treatment of atherosclerosis.

## Conclusions

γδ T cells orchestrate a multifaceted immune response in the context of atherosclerosis, with potential implications in either promoting or reducing the threats posed by atherosclerosis, depending on the specific subpopulations and their interactions within the high lipid environment. γδ T cells serve as a link between the innate and adaptive immune responses, potentially holding a pivotal role in the progression and regression of atherosclerosis, contingent on their energy requirements. Further research is warranted to unravel the precise roles and implications of different γδ T cell subsets in atherosclerosis. Such insights have the potential to unlock unique therapeutic strategies, including the induction of reparative γδ T cells through iPSC technology.

## Author contributions

LX: Conceptualization, Investigation, Writing – original draft, Writing – review & editing. FC: Conceptualization, Writing – original draft, Writing – review & editing. WF: Writing – original draft, Writing – review & editing. SS: Conceptualization, Writing – original draft, Writing – review & editing. DC: Conceptualization, Investigation, Software, Writing – original draft, Writing – review & editing.
